# Prenatal and postnatal small-quantity lipid-based nutrient supplements and children’s social–emotional difficulties at ages 9–11 y in Ghana: follow-up of a randomized controlled trial

**DOI:** 10.1016/j.ajcnut.2023.05.025

**Published:** 2023-05-29

**Authors:** Elizabeth L. Prado, Seth Adu-Afarwuah, Charles D. Arnold, Ebenezer Adjetey, Benjamin Amponsah, Helena Bentil, Kathryn G. Dewey, Amanda E. Guyer, Adom Manu, Mavis Mensah, Brietta M. Oaks, Maku Ocansey, Xiuping Tan, Paul D. Hastings

**Affiliations:** 1 Institute for Global Nutrition, Department of Nutrition, University of California–Davis, Davis, CA, USA; 2 Department of Nutrition and Food Science, University of Ghana, Accra, Ghana; 3 Psychology Department, University of Ghana, Accra, Ghana; 4 Center for Mind and Brain, University of California–Davis, Davis, CA, USA; 5 Department of Human Ecology, University of California–Davis, Davis, CA, USA; 6 Public Health, University of Ghana, Accra, Ghana; 7 Department of Nutrition and Food Sciences, University of Rhode Island, Kingston, RI, USA; 8 McKing Consulting Corporation; 9 Department of Psychology, University of California–Davis, Davis, CA, USA

**Keywords:** social–emotional development, lipid-based nutrient supplements, SQ-LNS, child development, Ghana

## Abstract

**Background:**

Provision of small-quantity lipid-based nutrient supplements (SQ-LNSs) during early life improves growth and development. In the International Lipid-Based Nutrient Supplements DYAD–Ghana trial, prenatal and postnatal SQ-LNS reduced social–emotional difficulties at age 5 y, with greater effects among children in less-enriched home environments.

**Objectives:**

We aimed to investigate the effect of prenatal and postnatal SQ-LNS on children's social–emotional problems at age 9–11 y.

**Methods:**

In 2009–2011, 1320 pregnant women ≤20 wk gestation were randomly assigned to receive the following daily until 6 mo postpartum: *1*) iron and folic acid until delivery, then placebo, *2*) multiple micronutrients (MMNs), or *3*) SQ-LNS (20 g/d). Children in group 3 received SQ-LNS from 6 to 18 mo. In 2021, we evaluated children's social–emotional outcomes with 6 assessment tools that used caregiver, teacher, and/or self-report to measure socioemotional difficulties, conduct problems, temperament, mood, anxiety, and emotion management.

**Results:**

We assessed outcomes in 966 children, comprising 79.4% of 1217 participants eligible for re-enrolment. No significant differences were found between the SQ-LNS and control (non-LNS groups combined) groups. Few children (<2%) experienced high parent-reported social–emotional difficulties at 9–11 y, in contrast to the high prevalence at age 5 in this cohort (25%). Among children in less-enriched early childhood home environments, the SQ-LNS group had 0.37 SD (−0.04 to 0.82) lower self-reported conduct problems than the control group (*P*-interaction = 0.047).

**Conclusions:**

Overall positive effects of SQ-LNS on social–emotional development previously found at age 5 y are not sustained to age 9–11 y; however, there is some evidence of positive effects among children in less-enriched environments. The lack of effects may be owing to low prevalence of social–emotional problems at preadolescence, resulting in little potential to benefit from early nutritional intervention at this age in this outcome domain. Follow-up during adolescence, when social-emotional problems more typically onset, may yield further insights.

This trial was registered at clinicaltrials.gov as NCT00970866. https://clinicaltrials.gov/ct2/show/record/NCT00970866

## Introduction

Millions of pregnant women and children globally consume inadequate diets, resulting in conditions among children such as stunting, wasting, anemia, micronutrient deficiencies, and poor cognitive, motor, and social-emotional development [1]. Adequate availability of nutrients is necessary for the neurodevelopmental processes that occur rapidly during pregnancy and infancy; therefore, improving maternal and child diets is essential to supporting children to fulfill their developmental potential [2]. Various strategies to improve maternal and child diets have been evaluated, such as supplementation with a single micronutrient (such as iron or vitamin A), supplementation with multiple micronutrients (MMNs) (such as a capsule or powder), and nutrition education to caregivers. Although some positive effects of such interventions have been shown [3], the strategy with the strongest evidence for concurrent positive effects on children’s health, growth, and development is provision of small-quantity lipid-based nutrient supplements (SQ-LNSs) to young children. SQ-LNSs are typically made from vegetable oil, peanut paste, milk powder, and sugar, with added vitamins and minerals, thus providing many of the micronutrients and fatty acids that are necessary for growth and brain development [4].

Meta-analyses of data from a large number of randomized trials and children (7 to 18 trials and 2700 to 41,000 children, depending on the outcome) have shown that providing SQ-LNS to children aged 6 to 24 mo resulted in better health, such as 27% reduction in mortality [5], 16% reduction in anemia, and 64% reduction in iron-deficiency anemia [6]; better growth, such as 12% reduction in stunting, 14% reduction in wasting, and 13% reduction in underweight [7]; and better development, such as 16% to 19% reduction in adverse language, motor, and social–emotional outcomes [8]. This is the first intervention for young children to show beneficial effects across these 4 crucial health outcome domains of growth, development, anemia, and mortality. Given the substantial positive effects evident at the end of the intervention period, the question remains whether there are sustained positive effects on outcomes in later childhood.

A follow-up study of the International Lipid-Based Nutrient Supplements trial in Ghana at age 4–6 y showed reduced social–emotional difficulties (−0.12 SD) in the group who was exposed to SQ-LNS (indirectly in utero and through breast milk before 6 mo of age, and directly at 6–18 mo of age), compared with control groups, with greater effects among children in less-enriched home environments (−0.22 SD) [9]. Of the nutrients provided by SQ-LNS, 2 potential candidates regarding biological explanations for the lower behavioral problem scores in the SQ-LNS group are the essential fatty acids provided by SQ-LNS (prenatally and postnatally) and the iron provided directly to children postnatally in the SQ-LNS group. Deficiencies in these nutrients have been associated with social–emotional regulation and behavioral problems during childhood [10,11]. In this study, we investigated whether the positive effects on social–emotional development observed at 4–6 y were sustained to age 9–11 y.

## Methods

### Objectives and hypotheses

Our objective was to investigate the effect of prenatal and postnatal SQ-LNS on social–emotional problems and self-regulation at age 9–11 y and in the context of children’s home environments. Our first hypothesis was that, compared with children who were not exposed to SQ-LNS in early life, those exposed to SQ-LNS would experience fewer social–emotional problems and better self-regulation. Our second hypothesis was that children in less-enriched home environments during early childhood would experience more social–emotional problems and worse self-regulation. Our third hypothesis was that greater effects of SQ-LNS would be found among children from less-enriched home environments.

### Participants and procedure

The methods of the International Lipid-Based Nutrient Supplements-DYAD–Ghana randomized controlled trial (RCT), performed in 2009–2014 [12,13], and the 5-y follow-up study performed in 2016 [9,14], have been described in detail. In brief, 1320 pregnant women in the Somanya-Odumase-Kpong area of Ghana, a semiurban area in the Yilo Krobo and Lower Manya Krobo districts, were randomly assigned to one of the 3 groups. The iron and folic acid (IFA) group was assigned to receive 1 capsule per day containing 60 mg iron and 400 μg folic acid from enrolment to delivery and a placebo capsule containing 200 mg calcium from delivery to 6 mo postpartum. The MMN group was assigned to receive 1 capsule per day from enrolment to 6 mo postpartum containing 20 mg iron and 400 μg folic acid plus 16 additional micronutrients (Table 1) [15]. The SQ-LNS group was assigned to receive daily sachets of SQ-LNS produced by Nutriset SAS (Malaunay, France) from enrolment to 6 mo postpartum. The daily dose (20 g) contained the same micronutrients as the MMN capsules, 4 additional minerals (Ca, K, Mg, and P), protein, fat, and 118 kcal (Table 1). From the 6 to 18 mo of age, children in the SQ-LNS group received 20 g per day of SQ-LNS with micronutrient composition designed for children (Table 1). Participants were aware whether they received SQ-LNS or a capsule but were blinded to whether they received IFA or MMN. Field workers who assessed outcomes and collected other data were blinded to all intervention groups. Ethical approval for the study procedures was obtained from the Ethics Committees at the University of California Davis and the Ghana Health Service. The protocol was registered at clinicaltrials.gov as NCT00970866.

**TABLE 1:** Nutrient and energy contents of the dietary supplements.

	IFA	MMN	Maternal SQ-LNS	Child SQ-LNS
Ration per day	1 capsule	1 capsule	20-g sachet	20-g sachet
Total energy (kcal)	0	0	118	118
Protein (g)	0	0	2.6	2.6
Fat (g)	0	0	10	9.6
Linoleic acid (g)	0	0	4.59	4.46
α-Linolenic acid (g)	0	0	0.59	0.58
Vitamin A (μg RE)	0	800	800	400
Vitamin C (mg)	0	100	100	30
Vitamin B-1 (mg)	0	2.8	2.8	0.3
Vitamin B-2 (mg)	0	2.8	2.8	0.4
Niacin (mg)	0	36	36	4
Folic acid (μg)	400	400	400	80
Pantothenic acid (mg)	0	7	7	1.8
Vitamin B-6 (mg)	0	3.8	3.8	0.3
Vitamin B-12 (μg)	0	5.2	5.2	0.5
Vitamin D (mg)	0	10	10	5
Vitamin E (mg)	0	20	20	6
Vitamin K (μg)	0	45	45	30
Iron (mg)	60	20	20	6
Zinc (mg)	0	30	30	8
Copper (mg)	0	4	4	0.34
Calcium (mg)	0	0	280	280
Phosphorus (mg)	0	0	190	190
Potassium (mg)	0	0	200	200
Magnesium (mg)	0	0	65	40
Selenium (μg)	0	130	130	20
Iodine (μg)	0	250	250	90
Manganese (mg)	0	2.6	2.6	1.2

IFA, iron and folic acid capsule; MMN, multiple micronutrient supplement capsule; SQ-LNS, small-quantity lipid-based nutrient supplement. Information from table previously published [15].

For the 10-y follow-up study, data collection was performed from December 2020 to December 2021, when children were aged 9–11 y. Field workers contacted caregivers of 1217 children eligible for re-enrolment, excluding those known to have not survived. At a home visit, field workers obtained informed consent, collected sociodemographic information, assessed the home environment, and scheduled the caregiver to bring the children to the project office. At the project office visit, field workers assessed children's health, anthropometric status, and cognitive development and obtained parent and child reports on child social–emotional and behavioral problems. Field workers also visited each child’s school to administer teacher report and school quality interviews.

On the basis of our successful follow-up of ∼1000 children at age 4–6 y, we expected to reenroll ∼900 at age 9–11 y. Because children in the 2 control groups (IFA and MMN) did not receive any nutrition supplements, the main goal of the follow-up assessments was to compare the lipid-based nutrient supplement (LNS) with non-LNS (IFA + MMN groups combined) groups. A sample size of 900 (300 LNS, 600 non-LNS) provides 80% power to detect an effect size, that is, a mean difference between 2 groups, of >0.20 SD for continuous outcomes at a significance level of *P* < 0.05. For the analysis of intervention effects among children exposed to less-enriched home environments, we determined the HOME score cutoff for subgroup analyses based on the regions of significance analysis, as described below. For subgroup analyses, an analysis using half of the target sample (150 LNS, 300 non-LNS) provided 80% power to detect an effect size of >0.28 SD, and using a quarter of the target sample (75 LNS, 150 non-LNS) provided 80% power to detect an effect size of >0.40 SD.

### Assessment of background characteristics and home environment

At enrolment into the parent trial, maternal and household information, such as maternal age, parity, education, and household assets, was collected by trained field workers using a questionnaire. Trained laboratory personnel measured maternal blood hemoglobin concentration at a clinic visit using a digital Hemocue (HemoCue model 301, AG, Switzerland). Infant weight and length were measured within 48 h of birth or between 3 and 14 d after birth for 87 children (9.4%) for whom the former was not possible.

At the 5-y follow-up, we assessed the home environment using the Early Childhood Home Observation for the Measurement of the Environment (EC-HOME) inventory [16], which we adapted to the local context in Ghana. The EC-HOME inventory is designed for children aged 3–6 y. Our adapted version contained 46 items measuring learning materials, language stimulation, physical environment, caregivers’ responsivity, academic stimulation, desirable behavior modeling, family lifestyle variety, and negative behavior acceptance. The adapted EC-HOME showed reasonable test–retest reliability (Pearson *r*) and internal reliability (Cronbach *α*) in the study setting (>0.63) [9].

At the 10-y follow-up, we assessed the home environment using the Middle Childhood version of the HOME inventory (MC-HOME), which is designed for use in children aged between 6 and 10 y. Our adapted version contained 58 items measuring parental responsivity, physical environment, learning materials, active stimulation, encouraging maturity, emotional climate, parental involvement, and family participation. We also assessed maternal depressive symptoms using the Center for Epidemiological Studies–Depression test [17] and pubertal development using the Petersen Pubertal Development Scale by parent and child report [18]. Both the MC-HOME and Center for Epidemiological Studies–Depression test showed high test–retest and internal reliability (>0.75) (Supplemental Table 1), whereas reliability was lower for reported pubertal development (0.51–0.70) (Supplemental Table 1).

### Assessment of outcomes

We assessed children social–emotional problems through the following tools: We administered the strengths and difficulties questionnaire (SDQ) by parent report, teacher report, and child self-report. A set of 20 questions rate children’s behavior during the past 6 mo in 4 subscales: emotional symptoms, conduct problems, hyperactivity/inattention, and peer relationship problems [19]. We administered the Brief Problem Monitor–Parent (BPM-P) by parent report, which is a set of 19 questions that measure attention problems, internalizing problems, and externalizing problems [20]. We administered the Mood and Feelings Questionnaire (MFQ) by child self-report, which is a set of 13 questions that rate mood and feelings during the past 2 wk [21]. We administered the Screen for Child Anxiety-Related Emotional Disorders (SCARED) by child self-report. The SCARED includes 41 questions designed to screen for signs of anxiety disorders in children, such as panic disorder, generalized anxiety disorder, separation anxiety, social anxiety disorder, and school avoidance; questions are combined into a total score [22]. For all these assessments, a higher score indicates higher problems.

We assessed child self-regulation through the following tools: We administered the Revised Early Adolescent Temperament Questionnaire (EATQ-P) by parent report [23], measuring activation control (the capacity to perform an action when there is a strong tendency to avoid it), attention (the capacity to focus attention and to shift attention when desired), and inhibitory control (the capacity to plan and to suppress inappropriate responses). For this tool, a higher score indicates higher self-regulation. We administered the Children’s Emotion Management Scales (CEMS) by child self-report, in which 9 items measure behaviorally oriented management of sadness, anger, and anxiety [24]. For this tool, a higher score indicates higher self-regulation problems.

Each item was translated into the local languages (Krobo, Ewe, and Twi) and backtranslated to English. If any discrepancies were found between the original item and the backtranslation, the translation was revised and backtranslation was repeated until the meanings matched. Adapted assessments were evaluated for test–retest reliability in a series of 2 pilot tests, each with a sample of 30 children aged 9–11 y in the study area. Assessments that showed low reliability were revised and reevaluated. Test–retest reliability (Pearson r) ranged from 0.45 to 0.83, with most scores (9/12) >0.65 (Supplemental Table 1). Internal reliability (Cronbach α) in the full sample ranged from 0.54 to 0.88, with 6 of the 12 assessments >0.7 and 2 assessments ∼0.7 (α = 0.67–0.68) (Supplemental Table 1). The remaining 4 scales with an α of 0.54–0.62 consisted of only 3 to 6 items, thus it is not surprising that Cronbach α was slightly lower given that it tends to be lower for scales with fewer items [25].

### Statistical analyses

A statistical analysis plan was posted to Open Science Framework before performing analyses (https://osf.io/bmv9d/). Analyses were conducted in R v4.1.1.

### Group characteristic comparisons

We examined whether children in the SQ-LNS and control groups were similar in key baseline characteristics by presenting descriptive statistics for each intervention group. To evaluate potential bias in the sample, we compared baseline characteristics between the sample included in the analysis and the sample enrolled in the parent trial but lost to follow-up, using *t* tests for continuous variables and χ^2^ tests for categorical variables.

### Effect of SQ-LNS

We conducted a complete case intention-to-treat analysis. To test hypothesis 1, for each of the 8 outcomes described earlier (SDQ-Child, SDQ-Parent, SDQ-Teacher, BPM-P, MFQ, SCARED, EATQ-P, and CEMS), we examined the difference between the intervention (SQ-LNS) and control (non-LNS) groups using multivariate analysis of covariance (MANCOVA) models for outcomes with multiple subscales and ANCOVA models for single scale outcomes. Our primary comparison of interest was the SQ-LNS group with the control group (IFA and MMN groups combined). However, we also compared the IFA and MMN groups to confirm that it was appropriate to combine them, as demonstrated by no significant difference at *P* > 0.10 in outcomes between those 2 groups. If the IFA and MMN groups were found to be different from one another, we conducted a 3-group comparison analysis and post hoc Tukey-Kramer pairwise comparisons. For outcomes with multiple subscale scores (SDQ: 4 subscales, EATQ: 3 subscales, CEMS: 3 subscales), we used the MANCOVA global effect test to determine the overall effect on the outcome and examined post hoc tests on each subscale score using the Holm–Bonferroni method to adjust for multiple testing within each model.

Three versions of each model were assessed. The first model was adjusted for child age at follow-up only. The second model was additionally adjusted for child sex, developmental assessment data collector, and any of the following baseline variables that were significantly associated (*P* < 0.10) with the outcome in the correlation analysis: maternal age, maternal education, maternal prepregnancy BMI, maternal hemoglobin concentration, household asset index, and parity. The third model was additionally adjusted for any factors collected after enrolment (birth weight) or at follow-up (school grade and quality, EC-HOME score, MC-HOME score, maternal depressive symptoms, and child pubertal development score) that were significantly associated (*P* < 0.10) with the outcome in the correlation analysis. For any covariates that were collected after baseline, we first determined whether they were different between the intervention groups at *P* < 0.10. If so, we did not include them in the model because they are potential mediators. Birth weight was excluded based on this criterion. If inclusion of covariates resulted in reduction in sample size of >10% owing to missing covariates, which was the case for the school quality indicators, we used simple imputation to fill in missing covariate values in model 3.

To test hypotheses 2 and 3, we added to models 1 and 3 the EC-HOME score at 5 y, then the interaction between the intervention group and EC-HOME scores. As reported previously, EC-HOME scores did not differ between the intervention groups [9]. Significant interactions (*P* < 0.10) were further examined with regions of significance analyses overall and for each subscale score [26]. For outcomes with multiple subscale scores, we used the MANCOVA global interaction test and examined post hoc tests for the interaction term within each subscale using the Holm–Bonferroni method to adjust for multiple testing within each model.

### Effect modifiers

In exploratory analyses, we evaluated potential effect modification by 5 additional variables: baseline household asset index, maternal education, prepregnancy BMI, primiparity, and child sex. We tested the interaction between each potential effect modifier and intervention groups. Significant interactions (*P* < 0.10) were further examined with regions of significance analyses [7].

## Results

**FIGURE 1:**
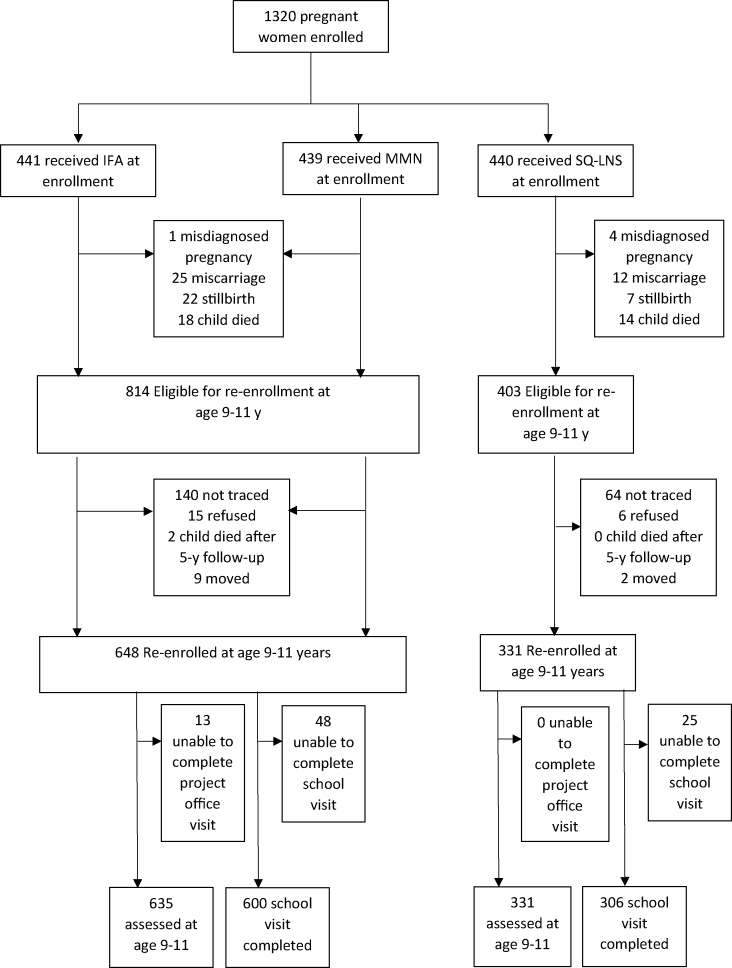
Trial profile. IFA, iron and folic acid; MMN, multiple micronutrient; SQ-LNS, small-quantity lipid-based nutrient supplement.

We successfully reenrolled 979 children at the home visit and collected data from 966 children at the project office visit, representing 107% of our target sample size (900), 73.2% of the 1320 pregnant women enrolled in the parent trial, and 79.4% of the 1217 children eligible for re-enrolment (excluding those known not to have survived). We successfully completed teacher interviews at school visits for 906 children. The proportion lost to follow-up was balanced between the intervention groups (*P* = 0.23) (Figure 1).

The 966 included in the analysis were similar to the 354 enrolled in the parent trial but lost to follow-up regarding most background characteristics, such as baseline maternal age, education, and BMI (Supplemental Table 2). However, those lost to follow up recorded lower gestational age at delivery (mean 38.9 vs. 39.3 wk) and a greater proportion of their mothers were nulliparous at enrolment (40% vs. 32%).

Among those included in the analysis, background characteristics were similar in the SQ-LNS and control groups (Table 2) [27]. In both groups, children were, on average, aged 9.9 y at follow-up assessment.

**TABLE 2:** Group characteristic comparisons.

Variable	SQ-LNS, *n* = 331∗	Control, *n* = 635∗
Baseline maternal age (y)	27.0 (5.5)	26.8 (5.4)
Prepregnancy BMI^1^ (kg/m^2^)	24.9 (4.5)	24.3 (4.4)
Gestational age at enrolment (wk)	16.0 (3.3)	16.2 (3.2)
Baseline maternal education (y)	7.6 (3.8)	7.7 (3.4)
Baseline maternal hemoglobin (g/L)	111 (11)	111 (13)
Baseline household asset index^2^	−0.07 (0.97)	0.08 (0.98)
Nulliparous (%)	30.8 [102/331]	31.8 [202/635]
Gestational age at delivery (wk)	39.3 (1.8)	39.3 (1.8)
Child male (%)	48.3 [160/331]	48.3 [307/635]
Child age at follow-up (y)	9.9 (0.5)	9.9 (0.5)
Mean maternal adherence from pregnancy through 6 mo postpartum (% of supplements consumed)	71.5 (19.4)	73.9 (22.0)
EC-HOME score at 5-y follow-up	28.0 (4.5)	28.0 (4.8)
MC-HOME score at 10-y follow-up	35.0 (5.5)	35.1 (6.1)

EC-HOME, Early Childhood Home Observation for the Measurement of the Environment Inventory; MC-HOME, Middle Childhood Home Observation for the Measurement of the Environment Inventory; SQ-LNS small-quantity lipid-based nutrient supplement.

∗ Values are given as mean (SD) or % [*n*/total]. Control refers to iron and folic acid + multiple micronutrient groups.

1 Estimated prepregnancy BMI was calculated from estimated prepregnancy weight (based on polynomial regression with gestational age, gestational age squared, and gestational age cubed as predictors) [27] and height at enrolment.

2 Proxy indicator for household socioeconomic status constructed for each household based on ownership of a set of assets (e.g., radio and television), lighting source, drinking water supply, sanitation facilities, and flooring materials. Household ownership of this set of assets is combined into an index (with a mean of zero and standard deviation of one) using the principal components analysis. A higher value represents a higher socioeconomic status.

### Effects of SQ-LNS on child social–emotional problems and self-regulation (hypothesis 1)

Children in the SQ-LNS group did not significantly differ from those in the combined control group in any outcome score, with mean differences ranging from −0.3 to 0.5 points and *P* values ranging from 0.14 to 0.99 (Table 3 and Supplemental Table 3). Children in the IFA and MMN groups did not significantly differ in any outcome, except that the IFA group showed lower internalizing problems (mean 2.21) than the MMN group (mean 2.52) on the BPM-P (Supplemental Table 4). However, the BPM-P scores were not significantly different in the 3-group comparison (*P* = 0.18) (Supplemental Table 5). At age 9–11 y, a small percentage (1.6%) of children had social–emotional difficulties scores in the high/very high range on the parent-reported SDQ (≥17), although the prevalence was higher for teacher-reported (11.6% with scores ≥16) and children self-reported (6.3% with scores ≥18) SDQ [28].

**TABLE 3:** Effect of SQ-LNS on each outcome score.

	SQ-LNS, mean (SD)	Control, mean (SD)	Model 1 adjusted *P*^1^	Model 3 adjusted *P*^2^
Strengths and Difficulties Questionnaire–Child (SDQ-C), overall MANCOVA *P*			0.895	0.954
SDQ-C emotional symptoms	3.58 (1.52)	3.58 (1.50)	0.999	0.999
SDQ-C conduct problems	1.90 (1.35)	1.94 (1.40)	0.999	0.999
SDQ-C hyperactivity/inattention	3.66 (1.52)	3.61 (1.71)	0.999	0.999
SDQ-C peer relationship problems	2.45 (1.25)	2.50 (1.31)	0.999	0.999
Strengths and Difficulties Questionnaire–Parent (SDQ-P), overall MANCOVA *P*			0.488	0.266
SDQ-P emotional symptoms	1.76 (1.40)	1.72 (1.43)	0.999	0.999
SDQ-P conduct problems	1.46 (1.29)	1.49 (1.38)	0.999	0.999
SDQ-P hyperactivity/inattention	2.77 (2.05)	2.74 (1.93)	0.999	0.999
SDQ-P peer relationship problems	1.28 (1.16)	1.41 (1.24)	0.399	0.102
Strengths and Difficulties Questionnaire–Teacher (SDQ-T), overall MANCOVA *P*			0.392	0.396
SDQ-T emotional symptoms	2.43 (2.10)	2.28 (2.12)	0.999	0.999
SDQ-T conduct problems	1.70 (1.84)	1.69 (1.89)	0.999	0.999
SDQ-T hyperactivity/inattention	3.18 (2.17)	3.37 (2.32)	0.999	0.384
SDQ-T peer relationship problems	1.76 (1.41)	1.82 (1.48)	0.999	0.999
Brief Problem Monitor–Parent (BPM-P), overall MANCOVA *P*			0.653	0.848
BPM-P internalizing problems total score	2.40 (1.69)	2.37 (1.68)	0.999	0.999
BPM-P externalizing problems	2.53 (2.16)	2.52 (2.23)	0.999	0.999
BPM-P attention problems	2.71 (2.12)	2.87 (2.18)	0.846	0.999
Mood and Feelings Questionnaire (MFQ) total score	2.05 (2.35)	2.00 (2.04)	0.766	0.799
Screen for Child Anxiety-Related Emotional Disorders (SCARED) total score	18.9 (5.8)	19.3 (6.0)	0.253	0.212
Revised Early Adolescent Temperament Questionnaire–Parent (EATQ-P), overall MANCOVA *P*			0.463	0.406
EATQ-P activation control subscale score	26.0 (5.4)	26.1 (5.5)	0.999	0.999
EATQ-P attention subscale score	22.2 (4.0)	22.0 (4.2)	0.999	0.999
EATQ-P inhibitory control subscale score	18.4 (3.9)	18.5 (4.0)	0.999	0.999
Children's Emotion Management Scales (CEMS), overall MANCOVA *P*			0.146	0.159
CEMS anger subscale score	1.46 (0.42)	1.51 (0.45)	0.202	0.211
CEMS sadness subscale score	1.47 (0.39)	1.52 (0.41)	0.202	0.211
CEMS worried subscale score	1.54 (0.47)	1.55 (0.48)	0.592	0.711

Control refers to iron and folic acid + multiple micronutrient groups. Results based on MANCOVA. For all assessments, a higher score indicates higher social–emotional problems, except the EATQ-P for which a higher score indicates a higher self-regulation.

SQ-LNS, small-quantity lipid-based nutrient supplement.

1 Model 1 was adjusted for child age at follow-up only. *P* values adjusted for multiple comparisons within scales based on the Holm–Bonferroni method. Model 2 results are reported in Supplemental Table 3.

2 Model 3 was additionally adjusted for child sex, developmental assessment data collector, and any of the following variables that were significantly associated at the *P* < 0.1 level with the outcome in the correlation analysis: baselin—maternal age, maternal education, maternal prepregnancy BMI, maternal hemoglobin concentration, household asset index, parity; and at the follow-up—school grade and quality, EC-HOME score, MC-HOME score, maternal depressive symptoms, and child pubertal development score. *P* values adjusted for multiple comparisons within scales based on the Holm–Bonferroni method.

### Association of home environment with children social–emotional problems and self-regulation (hypothesis 2)

Children with higher EC-HOME scores, indicating higher responsive care and learning opportunities in the home environment during early childhood, showed lower SDQ conduct problems (by parent and teacher reports) and lower SDQ hyperactivity/inattention (by parent report). They also demonstrated lower BPM-P externalizing and attention problems and higher EATQ-P self-regulation in attention and inhibitory control. EC-HOME scores were not significantly associated with self-report SDQ or MFQ scores or parent-report SCARED or CEMS scores (Table 4).

**TABLE 4:** Association of home environment scores with each outcome.^1^

	Model 1^2^		Model 3^3^		Model 3^3^	
	5-y EC-HOME		5-y EC-HOME		10-y MC-HOME	
	Estimate (SE)	*P*	Estimate (SE)	*P*	Estimate (SE)	*P*
Strengths and Difficulties Questionnaire–Child (SDQ-C)						
SDQ-C emotional symptoms	−0.01 (0.01)	0.374	-0.01 (0.01)	0.300	0.01 (0.01)	0.218
SDQ-C conduct problems	−0.01 (0.01)	0.334	0.00 (0.01)	0.992	−0.02 (0.01)	0.019
SDQ-C hyperactivity/inattention	−0.02 (0.01)	0.128	0.00 (0.01)	0.938	−0.03 (0.01)	0.016
SDQ-C peer relationship problems	−0.01 (0.01)	0.552	0.00 (0.01)	0.811	0.00 (0.01)	0.699
Strengths and Difficulties Questionnaire–Parent (SDQ-P)						
SDQ-P emotional symptoms	0.00 (0.01)	0.735	0.01 (0.01)	0.510	0.00 (0.01)	0.819
SDQ-P conduct problems	−0.04 (0.01)	<0.001	−0.03 (0.01)	0.008	−0.03 (0.01)	0.001
SDQ-P hyperactivity/inattention	−0.05 (0.01)	<0.001	−0.04 (0.02)	0.026	−0.03 (0.01)	0.011
SDQ-P peer relationship problems	−0.02 (0.01)	0.059	0.00 (0.01)	0.972	−0.02 (0.01)	0.002
Strengths and Difficulties Questionnaire–Teacher (SDQ-T)						
SDQ-T emotional symptoms	−0.02 (0.02)	0.231	−0.01 (0.02)	0.792	—	—
SDQ-T conduct problems	−0.03 (0.01)	0.032	−0.03 (0.02)	0.038	—	—
SDQ-T hyperactivity/inattention	−0.03 (0.02)	0.059	−0.02 (0.02)	0.280	—	—
SDQ-T peer relationship problems	0.00 (0.01)	0.693	0.00 (0.01)	0.728	—	—
Brief Problem Monitor–Parent (BPM-P)						
BPM-P internalizing problems total score	−0.01 (0.01)	0.455	−0.01 (0.01)	0.718	−0.01 (0.01)	0.491
BPM-P externalizing problems	−0.08 (0.02)	<0.001	−0.05 (0.02)	0.005	−0.05 (0.01)	0.001
BPM-P attention problems	−0.07 (0.02)	<0.001	−0.04 (0.02)	0.017	−0.05 (0.01)	0.001
Mood and Feelings Questionnaire (MFQ) total score	−0.02 (0.02)	0.321	−0.02 (0.02)	0.183	—	—
Screen for Child Anxiety-Related Emotional Disorders (SCARED) total score	−0.02 (0.04)	0.712	−0.05 (0.05)	0.256	0.10 (0.04)	0.005
Revised Early Adolescent Temperament Questionnaire–Parent (EATQ-P)						
EATQ-P activation control subscale score	0.14 (0.04)	0.001	0.06 (0.04)	0.178	0.10 (0.03)	0.003
EATQ-P attention subscale score	0.17 (0.03)	<0.001	0.10 (0.03)	0.002	0.09 (0.03)	0.001
EATQ-P inhibitory control subscale score	0.09 (0.03)	0.002	0.06 (0.03)	0.056	0.05 (0.03)	0.041
Children’s Emotion Management Scales (CEMS)						
CEMS anger subscale score	0.00 (0.00)	0.828	0.00 (0.00)	0.808	—	—
CEMS sadness subscale score	0.00 (0.00)	0.370	0.00 (0.00)	0.594	—	—
CEMS worried subscale score	0.00 (0.00)	0.946	0.00 (0.00)	0.681	—	—

EC-HOME, Early Childhood Home Observation for the Measurement of the Environment; MC-HOME, Middle Childhood Home Observation for the Measurement of the Environment.

1 Estimates are unstandardized *β* coefficients.

2 Model 1 was adjusted for the child age at the follow-up and for the intervention group only.

3 Model 3 was additionally adjusted for child sex, developmental assessment data collector, and any of the following variables that were significantly associated at the *P* < 0.1 level with the outcome in the correlation analysis: baseline—maternal age, maternal education, maternal prepregnancy BMI, maternal hemoglobin concentration, household asset index, and parity; and at the follow-up—school grade and quality, MC-HOME score, maternal depressive symptoms, and child pubertal development score.

MC-HOME scores were included as a covariate in model 3 if they were independently associated with the outcome at *P* < 0.10. When included, coefficients for MC-HOME scores were generally similar to or larger than coefficients for EC-HOME scores in model 3 (Table 4). Children with higher MC-HOME scores showed lower SDQ conduct problems (by self-report and parent report), lower SDQ hyperactivity/inattention (by self-report and parent report), and lower SDQ peer relationship problems (by the parent report). They also exhibited lower BPM-P externalizing and attention problems and higher EATQ-P self-regulation in activation control, attention, and inhibitory control. MC-HOME scores were not associated with teacher-report SDQ, self-report MFQ, or parent-report CEMS scores. The only result that was in the opposite direction, as expected, was that higher MC-HOME scores were associated with higher SCARED scores (anxiety). All but one of the prospective associations between EC-HOME scores and children's adjustment that were significant in model 1 remained significant in model 3, after including MC-HOME scores that were concurrent with children's adjustment measures.

### Interaction of SQ-LNS with home environment (hypothesis 3) and other potential effect modifiers

**FIGURE 2:**
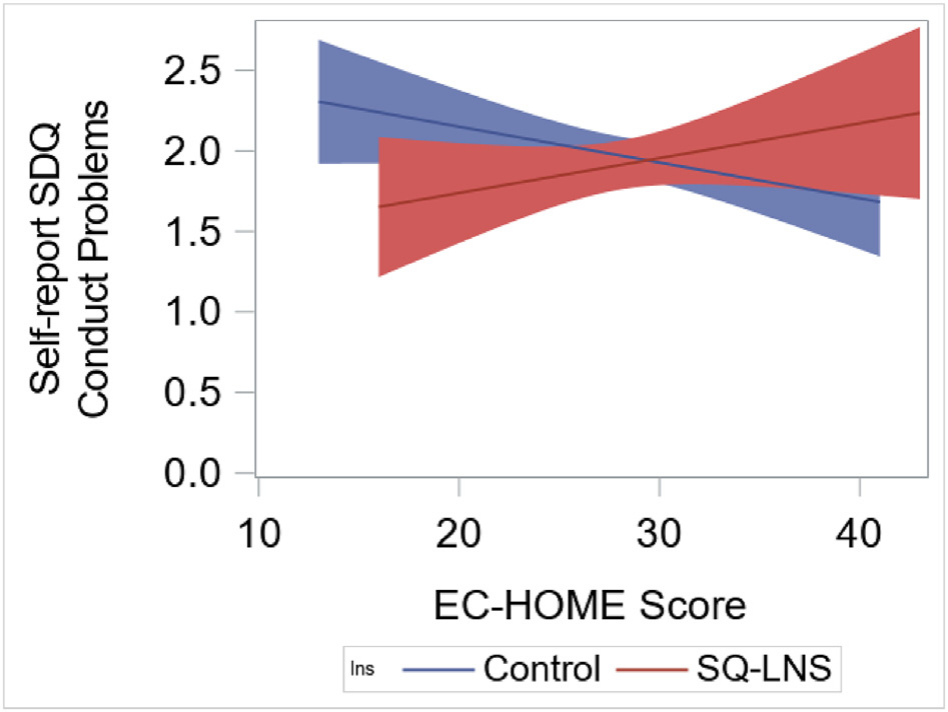
Association of SQ-LNS with self-reported SDQ conduct problems by Early Childhood Home Observation for the Measurement of the Environment (EC-HOME) scores. Among children with low EC-HOME scores (<23), the SQ-LNS group showed significantly lower conduct problems than the control group, whereas there were no significant differences between SQ-LNS and control groups amoung children with high EC-HOME scores. SDQ, strengths and difficulties questionnaire; SQ-LNS, small-quantity lipid-based nutrient supplement.

The interaction between the intervention group and EC-HOME score was significant for self-report SDQ conduct problems (*P*-interaction = 0.05) and hyperactivity/inattention (*P*-interaction = 0.08). For conduct problems, among children in less-enriched early childhood HOME environments (EC-HOME score of <23 based on the regions of significance analysis), the SQ-LNS group showed significantly lower conduct problems (mean = 1.67, SD = 1.49, *n* = 30) than the control group (mean = 2.26, SD = 1.61, *n* = 62), with a subgroup estimated standardized mean difference of 0.37 SD (95% CI, −0.04 to 0.82). Among children with high EC-HOME scores, there were no significant differences in self-report SDQ conduct problems between the intervention groups (Figure 2). For hyperactivity/inattention, the regions of significance analysis showed that there were no significant differences between the SQ-LNS and control groups at any values of EC-HOME scores.

**FIGURE 3:**
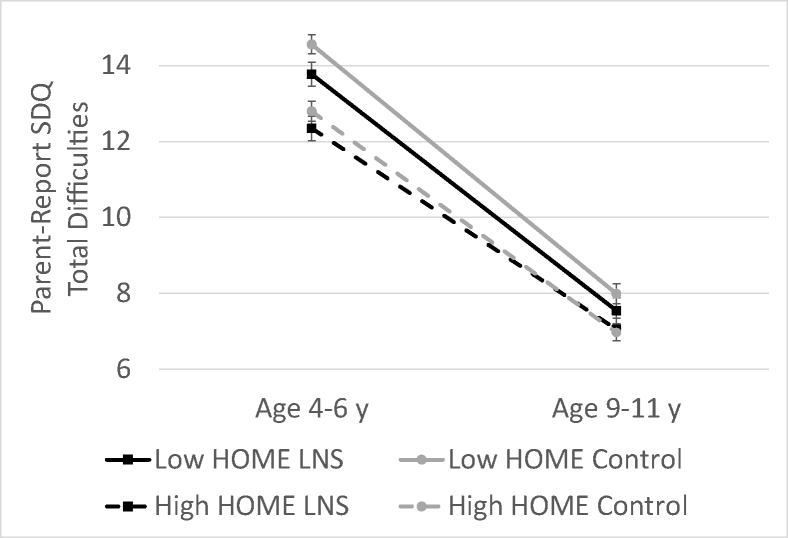
Total difficuties score on the parent-reported SDQ at age 4–6 y and 9–11 y by the intervention group and high compared with low EC-HOME scores. At the age 4–6 y, there were significantly greater effects of SQ-LNS among children with low HOME scores (less than the median). At the age 9–11 y, there were no signficiant differences between the groups. HOME, Home Observation for the Measurement of the Environment; LNS, lipid-based nutrient supplement; SDQ, strengths and difficulties questionnaire.

Because the parent-report SDQ was the only measure administered at both 4–6 y and 9–11 y, we examined the mean SDQ total difficulties scores at both time points by the intervention group and high compared with low EC-HOME scores (split at the median = 28) (Figure 3). At both time points, the rank order of the group means remained similar, with the lowest difficulties in the SQ-LNS group with high EC-HOME scores, followed by the control group with high EC-HOME scores, then the SQ-LNS group with low EC-HOME scores, and, finally, the control group with low EC-HOME scores. However, total mean difficulties declined over time in all groups and converged such that there were no significant intervention group differences in total difficulties at age 9–11 y.

For the exploratory effect modifiers, of the 115 interaction tests (23 outcomes by 5 exploratory effect modifiers), 14 (12%) were significant at *P* < 0.1, which is the number that would be expected by chance. Although the number of significant interactions was at chance levels, the pattern of findings for maternal education was consistent with the hypothesis of greater positive effects of SQ-LNS on child self-regulation in less-enriched home environments (i.e., those with lower maternal education). Details are reported in **Supplemental Results** and shown in Supplemental Figure 1.

## Discussion

In this long-term follow-up study of an RCT that provided a comprehensive daily nutritional supplement during most of the first 1000 d, from early pregnancy to 18 mo postpartum, we did not find effects on social–emotional problems at age 9–11 y. Thus, the previous finding showing reduced social–emotional difficulties in the SQ-LNS group at age 4–6 y was not sustained to age 9–11 y. We did find that children who experienced less-enriched early childhood home environments tended to have higher social–emotional difficulties at age 9–11 y for 8 of the 23 social–emotional scores examined. These associations were attenuated, but 7 remained significant, when adjusting for concurrent home environment, which was significantly associated with 11 of the 23 social–emotional scores. Among children who experienced less-enriched early childhood home environments, those in the SQ-LNS group recorded 0.37 SD lower self-reported conduct problems than those in the control group. This is consistent with the previous finding at age 4–6 y in the same cohort that SQ-LNS buffered children from social–emotional difficulties associated with less-enriched home environments.

To our knowledge, this is the first RCT in Africa to report a long-term follow-up assessment after nutritional supplementation with both macronutrients and micronutrients in both the prenatal and postnatal periods, which is a foundational period for brain development. We are aware of only 2 previous studies with a similar design, both of which were conducted in Latin America in the 1970s. One of these randomized only 4 villages to the intervention and control groups [29], and the other had a high rate of attrition, reenrolling only 55% of the original sample in the follow-up study [30,31]. Thus, our study, which was an individually randomized trial with a large sample size and high follow-up rate, makes an important contribution to the evidence for long-term effects of early nutrition on behavioral development. Our primary focus was on social–emotional outcomes due to the positive effects previously found at age 5 y. Effects on cognitive and academic skills, for which initial analyses were not significant [32], and other outcomes will be reported in future publications.

The lack of overall effects of SQ-LNS on child social–emotional problems may have been due to the low prevalence of social–emotional problems at 9–11 y of age, such that there was little potential to benefit from early nutritional intervention at this age in this outcome domain. Few children (<2%) experienced high social–emotional difficulties on the parent-reported SDQ at 9–11 y, in contrast to the high prevalence on this measure at age 5 y in the same cohort (25%). As shown in Figure 3, SDQ total difficulties declined over time in all intervention and home environment groups and converged such that there were no significant group differences at age 9–11 y. However, group means remained in the same order that was observed at age 4–6 y, with the highest difficulties in the control group with low EC-HOME scores. Adolescence is a period of high susceptibility to the onset of mental health problems, such as depressive and anxiety disorders and impulse control disorders [33]. At the next follow-up assessment at 11–13 y, which is currently in progress, we may observe an increase in social–emotional problems and divergence of group means, such that intervention effects may be easier to detect.

The finding that a less-enriched home environment during both early and middle childhood was associated with higher child social–emotional problems is consistent with findings among children in high-income countries [34,35]. A higher score on the HOME inventory reflects multiple aspects of the home environment, such as higher learning opportunities, parental responsivity and involvement, family participation, and a positive emotional climate. Our findings suggest that these aspects of the home environment are important for social–emotional development in diverse contexts, given that the setting and culture in Ghana is different from high-income countries where most research on social–emotional development has been conducted. These aspects of the home environment were associated with lower child behavior problems, particularly externalizing problems, such as conduct problems and hyperactivity/inattention, but not internalizing problems, such as mood symptoms and anxiety. The finding that both early childhood and preadolescent home environment scores were associated with behavior problems suggests that both early childhood and preadolescence are important periods to promote positive home environments for children's social–emotional well-being.

Our hypothesis that greater effects of SQ-LNS would be found among children from less-enriched home environments was partly supported by the interaction found for self-report conduct problems. Among children in less-enriched early childhood home environments, those in the SQ-LNS group scored substantially lower than the control group (0.37 SD) in this domain. This pattern is consistent with developmental theories that posit that children who experience biological vulnerabilities may show similar outcomes compared with children without biological vulnerabilities when raised in positive environments but may falter under conditions of environmental stress [36]. This finding should be interpreted with caution because this pattern was found for only one of the outcomes we examined. However, the robustness of the finding is supported by the consistency with the pattern found for social–emotional difficulties at age 4–6 y and the consistency of the pattern of the group means over time (Figure 2). Follow-up at future time points will provide further evidence to examine this hypothesis.

Strengths of the study were the individually randomized design, large number of children assessed 10 y after the trial was completed, balance of background characteristics between the intervention groups, a comprehensive assessment of various types of social–emotional problems using multiple tools and multiple respondents (self, parent, and teacher), and demonstrated reliability of the translated tools in the local context. One limitation was that those lost to follow-up differed significantly from those included in the analysis in gestational age at delivery and maternal parity. However, it is unlikely that this led to bias in the results because the difference in gestational age was small (38.9 vs. 39.3 wk) and there was no interaction between the intervention group and parity for most outcomes. Another limitation was that the minimum effect size we were able to detect with our sample size was 0.2 SD, whereas previous studies have shown positive effects of nutritional supplementation on developmental outcomes of ∼0.1 SD [37]. Thus, we may have missed significant effects owing to a lack of power to detect smaller differences between the groups.

Our study adds to the evidence base for the effectiveness of nutritional interventions such as SQ-LNS during the prenatal and postpartum periods. Although we did not find long-term effects on children's social–emotional problems, we found some evidence that there may be a positive effect among children in less-enriched home environments. Future work will examine other outcomes at this age, such as direct assessments of child behavior and physiology, and will follow up the cohort later in adolescence.

## Acknowledgments

We thank the families, teachers, and schools who participated in and supported this follow-up study and the Assistant Data Manager, Richard Azumah, for his contribution to data collection and cleaning. The authors’ responsibilities were as follows—KGD, SAA, ELP, PDH, AEG, AM, BMO: designed the research; SAA, EA, HB, MM, MO: conducted the research; CDA, XT, ELP: analyzed the data; ELP: wrote the paper, with critical input and comments from all other authors; ELP: had primary responsibility for final content; and all authors: have read and approved the final manuscript. The authors report no conflicts of interest.

### Funding

This publication is based on research funded by grants to the University of California, Davis, from the National Institutes of Health (R01HD099811) and the Bill & Melinda Gates Foundation (OPP49817). The funders did not play a role in the design or implementation of the study or interpretation of the data. The findings and conclusions contained within are those of the authors and do not necessarily reflect positions or policies of the Bill & Melinda Gates Foundation.

### Data availability

Deidentified individual participant data (such as data dictionaries) will be made available on request to researchers who provide a methodologically sound proposal and statistical analysis plan contingent on approval by the principal investigators. Proposals should be submitted to the corresponding author.

## Appendix A Supplementary data

Multimedia component1 Multimedia component1
