# Supplementary material for: Prenatal and postnatal small-quantity lipid-based nutrient supplements and children’s social–emotional difficulties at ages 9–11 y in Ghana: follow-up of a randomized controlled trial
Source: Am J Clin Nutr. 2023 May 29;118(2):433–42. doi: 10.1016/j.ajcnut.2023.05.025 (PMC10447494; doi:10.1016/j.ajcnut.2023.05.025)

Supplemental Material for the Manuscript “Pre- and postnatal small quantity lipid-based nutrient supplements and children’s social-emotional difficulties at age 9-11 years in Ghana: Follow-up of a randomized controlled trial” by Elizabeth L. Prado et al.

Supplemental Methods

*Effect modifiers*

In exploratory analyses, we evaluated potential effect modification by 5 additional variables: baseline household asset index, maternal education, pre-pregnancy BMI, primiparous, and child sex. We selected these effect modifiers for the following reasons. Similar to the HOME score, low household assets and maternal education may indicate a high risk home environment, therefore we expected greater effects of SQ-LNS in these groups. Effects of SQ-LNS on child growth status at birth were found among primiparous women in the parent study, therefore we expected that there may be longer-term positive effects in this group. Effects of SQ-LNS on growth status at age 4-6 years were found among women who were not overweight or obese at enrollment, therefore we also expected that there may be long-term positive effects in this group.

Supplemental Results

*Effect modifiers*

For the exploratory effect modifiers, out of 115 interaction tests (23 outcomes by 5 exploratory effect modifiers), 14 (12%) were significant at p < 0.1, which is the number that would be expected by chance. For self-report SDQ hyperactivity/inattention, the interaction of intervention group with household asset index was significant, however, regions of significance analysis showed no differences between groups. For parent-report SDQ conduct problems, the interaction of intervention group with maternal education was significant (p-interaction=0.09), however, regions of significance analysis showed no differences between groups. Compared to the control group, the SQ-LNS group had lower BPM-P externalizing problems (p-interaction=0.008) and CEMS self- regulation problems for anger (p-interaction=0.08) among children of mothers with low education, with significant differences for externalizing problems below 3.6 years of education and significant differences for anger regulation below 7.2 years of education. The control group had significantly lower BPM-P externalizing problems among children of mothers with education above 11.8 years (Supplemental Figure 1).

The interaction between intervention group and pre-pregnancy BMI was significant for four outcomes: self (p-interaction=0.01) and parent-report (p-interaction=0.07) SDQ hyperactivity/inattention, teacher-report SDQ emotional symptoms (p-interaction = 0.08), and EATQ inhibitory control (p-interaction = 0.09). The SQ-LNS group had significantly lower self-report hyperactivity/inattention among mothers with BMI above 30.5, while the control group had significantly lower hyperactivity/inattention among mothers with BMI below 19.5 (Supplemental Figure 1). Regions of significance analysis did not show any significant differences between groups for the other three outcomes.

The interaction between intervention group and parity was also significant for four outcomes: self-report SDQ conduct problems (p-interaction=0.09), parent-report SDQ emotional symptoms (p-interaction=0.08), and CEMS anger (p-interaction = 0.02) and sadness regulation (p-interaction=0.03). For both anger and sadness regulation, the SQ-LNS group had lower emotion regulation problems than the control group among multiparous women (Supplemental Figure 1). No significant group differences were found for the other two outcomes.

The interaction between intervention group and child sex was significant for the SCARED score (p-interaction=0.01) and CEMS anger regulation problems (p-interaction=0.04). For both outcomes, the SQ-LNS group had lower problems among males, but no differences among females (Supplemental Figure 1).

References

1. Cortina JM. What is coefficient alpha? An examination of theory and applications. Journal of Applied Psychology 1993;78:98-104. doi: 10.1037/0021-9010.78.1.98.

2. Adu-Afarwuah S, Lartey A, Okronipa H, Ashorn P, Ashorn U, Zeilani M, Arimond M, Vosti SA, Dewey KG. Maternal Supplementation with Small-Quantity Lipid-Based Nutrient Supplements Compared with Multiple Micronutrients, but Not with Iron and Folic Acid, Reduces the Prevalence of Low Gestational Weight Gain in Semi-Urban Ghana: A Randomized Controlled Trial. The Journal of nutrition 2017;147(4):697-705. doi: 10.3945/jn.116.242909.

*Supplemental Table 1*. Test-retest and internal reliability of social-emotional and other assessments.

|  |  | Number of items^1^ | Internal reliability: Cronbach’s Alpha  (n = 842-966) | Test-retest reliability: Pearson’s r  (n = 30) |
| --- | --- | --- | --- | --- |
| Caregiver report | |  |  |  |
|  | Strengths and Difficulties Questionnaire, Total Difficulties | 20 | 0.68 | 0.75 |
|  | Brief Problem Monitor, Parent (BPM-P) | 19 | 0.72 | 0.80 |
|  | Revised Early Adolescent Temperament Questionnaire |  |  |  |
|  | Activation Control | 7 | 0.72 | 0.73 |
|  | Attention | 6 | 0.54 | 0.72 |
|  | Inhibitory Control | 5 | 0.62 | 0.45 |
|  | Petersen Pubertal Development Scale | 10 | 0.52 | 0.70 |
| Teacher report | |  |  |  |
|  | Strengths and Difficulties Questionnaire, Total Difficulties | 20 | 0.77 | 0.72 |
| Child report | |  |  |  |
|  | Strengths and Difficulties Questionnaire, Total Difficulties | 20 | 0.75 | 0.55 |
|  | Mood and Feelings Questionnaire (MFQ) | 13 | 0.67 | 0.74 |
|  | Screen for Child Anxiety Related Emotional Disorders (SCARED) | 41 | 0.75 | 0.73 |
|  | Children's Emotion Management Scales (CEMS) |  |  |  |
|  | Anger | 3 | 0.61 | 0.66 |
|  | Sad | 3 | 0.55 | 0.63 |
|  | Worried | 3 | 0.74 | 0.68 |
|  | Petersen Pubertal Development Scale | 10 | 0.51 | 0.68 |
| Middle Childhood Home Observation for the Measurement of the Environment | | 58 | 0.77 | 0.86 |
| Maternal Depressive Symptoms: Center for Epidemiological Studies Depression Test | | 20 | 0.88 | 0.97 |

^1^Note that Cronbach’s Alpha tends to be lower for scales with a smaller number of items (1)

*Supplemental Table 2.* Comparison of characteristics between those included in the 10-year follow-up study versus those lost to follow-up

|  | | | |
| --- | --- | --- | --- |
| **Variable** | **Included**  n=966 | **Excluded**  n=354 |  |
|  | Mean (SD) or  % [n/total] | Mean (SD) or  % [n/total] | P-value |
| Baseline maternal age (y) | 26.9 (5.4) | 26.2 (5.7) | 0.062 |
| Pre-pregnancy BMI^1^ (kg/m^2^) | 24.5 (4.4) | 24.5 (4.2) | 0.979 |
| Gestational age at enrolment (wk) | 16.1 (3.2) | 16.1 (3.4) | 0.934 |
| Baseline maternal education (y) | 7.7 (3.6) | 7.5 (3.9) | 0.519 |
| Baseline maternal hemoglobin (g/L) | 111 (12) | 111 (12) | 0.845 |
| Baseline household asset index^2^ | 0.03 (0.97) | -0.08 (1.06) | 0.082 |
| Nulliparous at enrolment (%) | 31.5 [304/966] | 40.1 [142/354] | 0.003 |
| Gestational age at delivery (wk) | 39.3 (1.8) | 38.9 (2.6) | 0.004 |
| Child male (%) | 48.3 [467/966] | 51.6 [146/283] | 0.337 |

Results are based on ANOVA or Chi-square.

^1^Estimated pre-pregnancy BMI was calculated from estimated pre-pregnancy weight (based on polynomial regression with gestational age, gestational age squared, and gestational age cubed as predictors) (2) and height at enrollment.

^2^Proxy indicator for household socioeconomic status constructed for each household based on ownership of a set of assets (radio, television etc.), lighting source, drinking water supply, sanitation facilities, and flooring materials. Household ownership of this set of assets is combined into an index (with a mean of zero and standard deviation of one) using principal components analysis. Higher value represents higher socioeconomic status.

*Supplemental Table 3.* Effect of SQ-LNS on each outcome score in Models 1, 2, and 3

|  |  | SQ-LNS | Control | Model 1^1^ | | Model 2^2^ | | Model 3^3^ | |
| --- | --- | --- | --- | --- | --- | --- | --- | --- | --- |
|  |  | Mean (SD) | Mean (SD) | Unadjusted p-value | Holm-Bonferroni adjusted p-value | Unadjusted p-value | Holm-Bonferroni adjusted p-value | Unadjusted p-value | Holm-Bonferroni adjusted p-value |
| Strengths and Difficulties Questionnaire-Child (SDQ-C) overall MANCOVA p-value | |  |  | 0.895 |  | 0.954 |  | 0.954 |  |
|  | SDQ-C emotional symptoms | 3.58 (1.52) | 3.58 (1.50) | 0.965 | 0.999 | 0.695 | 0.999 | 0.693 | 0.999 |
|  | SDQ-C conduct problems | 1.90 (1.35) | 1.94 (1.40) | 0.624 | 0.999 | 0.565 | 0.999 | 0.563 | 0.999 |
|  | SDQ-C hyperactivity/inattention | 3.66 (1.52) | 3.61 (1.71) | 0.686 | 0.999 | 0.952 | 0.999 | 0.953 | 0.999 |
|  | SDQ-C peer relationship problems | 2.45 (1.25) | 2.50 (1.31) | 0.588 | 0.999 | 0.534 | 0.999 | 0.535 | 0.999 |
| Strengths and Difficulties Questionnaire-Parent (SDQ-P) overall MANCOVA p-value | |  |  | 0.488 |  | 0.431 |  | 0.266 |  |
|  | SDQ-P emotional symptoms | 1.76 (1.40) | 1.72 (1.43) | 0.695 | 0.999 | 0.804 | 0.999 | 0.662 | 0.999 |
|  | SDQ-P conduct problems | 1.46 (1.29) | 1.49 (1.38) | 0.704 | 0.999 | 0.327 | 0.982 | 0.369 | 0.999 |
|  | SDQ-P hyperactivity/inattention | 2.77 (2.05) | 2.74 (1.93) | 0.835 | 0.999 | 0.918 | 0.999 | 0.722 | 0.999 |
|  | SDQ-P peer relationship problems | 1.28 (1.16) | 1.41 (1.24) | 0.100 | 0.399 | 0.069 | 0.276 | 0.025 | 0.102 |
| Strengths and Difficulties Questionnaire-Teacher (SDQ-T) overall MANCOVA p-value | |  |  | 0.392 |  | 0.453 |  | 0.396 |  |
|  | SDQ-T emotional symptoms | 2.43 (2.10) | 2.28 (2.12) | 0.313 | 0.999 | 0.452 | 0.999 | 0.703 | 0.999 |
|  | SDQ-T conduct problems | 1.70 (1.84) | 1.69 (1.89) | 0.937 | 0.999 | 0.923 | 0.999 | 0.642 | 0.999 |
|  | SDQ-T hyperactivity/inattention | 3.18 (2.17) | 3.37 (2.32) | 0.258 | 0.999 | 0.188 | 0.752 | 0.096 | 0.384 |
|  | SDQ-T peer relationship problems | 1.76 (1.41) | 1.82 (1.48) | 0.570 | 0.999 | 0.619 | 0.999 | 0.447 | 0.999 |
| Brief Problem Monitor-Parent (BPM-P) overall MANCOVA p-value | |  |  | 0.653 |  | 0.689 |  | 0.848 |  |
|  | BPM-P internalizing problems total score | 2.40 (1.69) | 2.37 (1.68) | 0.791 | 0.999 | 0.614 | 0.999 | 0.763 | 0.999 |
|  | BPM-P externalizing problems | 2.53 (2.16) | 2.52 (2.23) | 0.999 | 0.999 | 0.797 | 0.999 | 0.931 | 0.999 |
|  | BPM-P attention problems | 2.71 (2.12) | 2.87 (2.18) | 0.282 | 0.846 | 0.305 | 0.916 | 0.441 | 0.999 |
| Mood and Feelings Questionnaire (MFQ) total score | | 2.05 (2.35) | 2.00 (2.04) | 0.766 | 0.766 | 0.784 | 0.784 | 0.799 | 0.799 |
| Screen for Child Anxiety Related Emotional Disorders (SCARED) total score | | 18.9 (5.8) | 19.3  (6.0) | 0.253 | 0.253 | 0.201 | 0.201 | 0.212 | 0.212 |
| Revised Early Adolescent Temperament Questionnaire-Parent (EATQ-P) overall MANCOVA p-value | |  |  | 0.463 |  | 0.358 |  | 0.406 |  |
|  | EATQ-P activation control subscale score | 26.0 (5.4) | 26.1 (5.5) | 0.691 | 0.999 | 0.780 | 0.999 | 0.794 | 0.999 |
|  | EATQ-P attention subscale score | 22.2 (4.0) | 22.0 (4.2) | 0.376 | 0.999 | 0.258 | 0.773 | 0.437 | 0.999 |
|  | EATQ-P inhibitory control subscale score | 18.4 (3.9) | 18.5 (4.0) | 0.720 | 0.999 | 0.727 | 0.999 | 0.374 | 0.999 |
| Children's Emotion Management Scales (CEMS) overall MANCOVA p-value | |  |  | 0.146 |  | 0.178 |  | 0.159 |  |
|  | CEMS anger subscale score | 1.46 (0.42) | 1.51 (0.45) | 0.078 | 0.202 | 0.088 | 0.263 | 0.070 | 0.211 |
|  | CEMS sadness subscale score | 1.47 (0.39) | 1.52 (0.41) | 0.067 | 0.202 | 0.107 | 0.263 | 0.103 | 0.211 |
|  | CEMS worried subscale score | 1.54 (0.47) | 1.55 (0.48) | 0.592 | 0.592 | 0.737 | 0.737 | 0.711 | 0.711 |

BMI = body mass index. SQ-LNS = Small Quantity Lipid-based Nutrient Supplement. Control = Iron & folic acid + multiple micronutrient groups. Results based on MANCOVA.

^1^Model 1 was adjusted for child age at follow-up only.

^2^Model 2 was additionally adjusted for child sex, developmental assessment data collector, and any of the following baseline variables that were significantly associated at the P < 0.1 level with the outcome in correlation analysis: maternal age, maternal education, maternal pre-pregnancy BMI, maternal hemoglobin concentration, household asset index, and parity.

^3^Model 3 was additionally adjusted for any of the following variables collected at follow-up that were significantly associated at the P < 0.1 level with the outcome in correlation analysis: school grade and quality, EC-HOME score, MC-HOME score, maternal depressive symptoms, and child pubertal development score.

*Supplemental Table 4.* Mean differences in each outcome score between the two control groups (IFA and MMN)

|  |  | Iron and Folic Acid (IFA) | Multiple Micronutrients (MMN) | Model 1^1^ | |
| --- | --- | --- | --- | --- | --- |
|  |  | Mean (SD) | Mean (SD) | Unadjusted p-value | Holm-Bonferroni adjusted p-value |
| Strengths and Difficulties Questionnaire-Child (SDQ-C) overall MANCOVA p-value | |  |  | 0.782 |  |
|  | SDQ-C emotional symptoms | 3.61 (1.48) | 3.56 (1.52) | 0.740 | 0.999 |
|  | SDQ-C conduct problems | 1.91 (1.37) | 1.97 (1.43) | 0.581 | 0.999 |
|  | SDQ-C hyperactivity/inattention | 3.59 (1.69) | 3.63 (1.73) | 0.761 | 0.999 |
|  | SDQ-C peer relationship problems | 2.44 (1.28) | 2.56 (1.34) | 0.268 | 0.999 |
| Strengths and Difficulties Questionnaire-Parent (SDQ-P) overall MANCOVA p-value | |  |  | 0.191 |  |
|  | SDQ-P emotional symptoms | 1.74 (1.40) | 1.69 (1.46) | 0.641 | 0.999 |
|  | SDQ-P conduct problems | 1.40 (1.37) | 1.58 (1.38) | 0.083 | 0.330 |
|  | SDQ-P hyperactivity/inattention | 2.79 (2.01) | 2.69 (1.86) | 0.522 | 0.999 |
|  | SDQ-P peer relationship problems | 1.39 (1.25) | 1.44 (1.23) | 0.526 | 0.999 |
| Strengths and Difficulties Questionnaire-Teacher (SDQ-T) overall MANCOVA p-value | |  |  | 0.106 |  |
|  | SDQ-T emotional symptoms | 2.06 (2.01) | 2.51 (2.21) | 0.008 | 0.033 |
|  | SDQ-T conduct problems | 1.70 (1.99) | 1.69 (1.79) | 0.925 | 0.999 |
|  | SDQ-T hyperactivity/inattention | 3.38 (2.35) | 3.36 (2.30) | 0.920 | 0.999 |
|  | SDQ-T peer relationship problems | 1.79 (1.54) | 1.84 (1.42) | 0.669 | 0.999 |
| Brief Problem Monitor-Parent (BPM-P) overall MANCOVA p-value | |  |  | 0.044 |  |
|  | BPM-P internalizing problems total score | 2.21 (1.64) | 2.52 (1.70) | 0.018 | 0.053 |
|  | BPM-P externalizing problems | 2.52 (2.28) | 2.52 (2.17) | 0.934 | 0.934 |
|  | BPM-P attention problems | 2.97 (2.23) | 2.77 (2.11) | 0.237 | 0.475 |
| Mood and Feelings Questionnaire (MFQ) total score | | 2.05 (2.05) | 1.95 (2.04) | 0.568 | - |
| Screen for Child Anxiety Related Emotional Disorders (SCARED) total score | | 19.4 (6.1) | 19.2 (5.9) | 0.658 | - |
| Revised Early Adolescent Temperament Questionnaire-Parent (EATQ-P) overall MANCOVA p-value | |  |  | 0.355 |  |
|  | EATQ-P activation control subscale score | 25.8 (5.4) | 26.5 (5.5) | 0.107 | 0.322 |
|  | EATQ-P attention subscale score | 21.7 (4.1) | 22.3 (4.3) | 0.132 | 0.322 |
|  | EATQ-P inhibitory control subscale score | 18.3 (4.1) | 18.8 (4.0) | 0.167 | 0.322 |
| Children's Emotion Management Scales (CEMS) overall MANCOVA p-value | |  |  | 0.799 |  |
|  | CEMS anger subscale score | 1.52 (0.45) | 1.51 (0.45) | 0.775 | 0.999 |
|  | CEMS sadness subscale score | 1.53 (0.41) | 1.51 (0.40) | 0.430 | 0.999 |
|  | CEMS worried subscale score | 1.55 (0.50) | 1.55 (0.46) | 0.946 | 0.999 |

^1^Model 1 was adjusted for child age at follow-up only.

*Supplemental Table 5*. Comparison between three intervention groups (IFA, MMN, and LNS) for the Brief Problem Monitor scale

|  |  | Iron and Folic Acid (IFA) | Multiple Micronutrients (MMN) | SQ-LNS | Model 1^1^ | |
| --- | --- | --- | --- | --- | --- | --- |
|  |  | Mean (SD) | Mean (SD) | Mean (SD) | Unadjusted p-value | Holm-Bonferroni adjusted p-value |
| Strengths and Difficulties Questionnaire-Child (SDQ-C) overall MANCOVA p-value | |  |  |  | 0.944 |  |
|  | SDQ-C emotional symptoms | 3.61 (1.48) | 3.56 (1.52) | 3.58 (1.52) | 0.951 | 0.999 |
|  | SDQ-C conduct problems | 1.91 (1.37) | 1.97 (1.43) | 1.90 (1.35) | 0.757 | 0.999 |
|  | SDQ-C hyperactivity/inattention | 3.59 (1.69) | 3.63 (1.73) | 3.66 (1.52) | 0.880 | 0.999 |
|  | SDQ-C peer relationship problems | 2.44 (1.28) | 2.56 (1.34) | 2.45 (1.25) | 0.462 | 0.999 |
| Strengths and Difficulties Questionnaire-Parent (SDQ-P) overall MANCOVA p-value | |  |  |  | 0.293 |  |
|  | SDQ-P emotional symptoms | 1.74 (1.40) | 1.69 (1.46) | 1.76 (1.40) | 0.829 | 0.999 |
|  | SDQ-P conduct problems | 1.40 (1.37) | 1.58 (1.38) | 1.46 (1.29) | 0.192 | 0.768 |
|  | SDQ-P hyperactivity/inattention | 2.79 (2.01) | 2.69 (1.86) | 2.77 (2.05) | 0.807 | 0.999 |
|  | SDQ-P peer relationship problems | 1.39 (1.25) | 1.44 (1.23) | 1.28 (1.16) | 0.209 | 0.768 |
| Strengths and Difficulties Questionnaire-Teacher (SDQ-T) overall MANCOVA p-value | |  |  |  | 0.160 |  |
|  | SDQ-T emotional symptoms | 2.06 (2.01) | 2.51 (2.21) | 2.43 (2.10) | 0.018 | 0.074 |
|  | SDQ-T conduct problems | 1.70 (1.99) | 1.69 (1.79) | 1.70 (1.84) | 0.991 | 0.999 |
|  | SDQ-T hyperactivity/inattention | 3.38 (2.35) | 3.36 (2.30) | 3.18 (2.17) | 0.524 | 0.999 |
|  | SDQ-T peer relationship problems | 1.79 (1.54) | 1.84 (1.42) | 1.76 (1.41) | 0.785 | 0.999 |
| Brief Problem Monitor-Parent (BPM-P) overall MANCOVA p-value | |  |  |  | 0.142 |  |
|  | BPM-P internalizing problems total score | 2.21 (1.64) | 2.52 (1.70) | 2.40 (1.69) | 0.059 | 0.177 |
|  | BPM-P externalizing problems | 2.52 (2.28) | 2.52 (2.17) | 2.53 (2.16) | 0.997 | 0.997 |
|  | BPM-P attention problems | 2.97 (2.23) | 2.77 (2.11) | 2.71 (2.12) | 0.276 | 0.552 |
| Mood and Feelings Questionnaire (MFQ) total score | | 2.05 (2.05) | 1.95 (2.04) | 2.05 (2.35) | 0.588 | - |
| Screen for Child Anxiety Related Emotional Disorders (SCARED) total score | | 19.4 (6.1) | 19.2 (5.9) | 18.9 (5.8) | 0.227 | - |
| Revised Early Adolescent Temperament Questionnaire-Parent (EATQ-P) overall MANCOVA p-value | |  |  |  | 0.440 |  |
|  | EATQ-P activation control subscale score | 25.8 (5.4) | 26.5 (5.5) | 26.0 (5.4) | 0.257 | 0.636 |
|  | EATQ-P attention subscale score | 21.7 (4.1) | 22.3 (4.3) | 22.2 (4.0) | 0.212 | 0.636 |
|  | EATQ-P inhibitory control subscale score | 18.3 (4.1) | 18.8 (4.0) | 18.4 (3.9) | 0.354 | 0.636 |
| Children's Emotion Management Scales (CEMS) overall MANCOVA p-value | |  |  |  | 0.378 |  |
|  | CEMS anger subscale score | 1.52 (0.45) | 1.51 (0.45) | 1.46 (0.42) | 0.203 | 0.411 |
|  | CEMS sadness subscale score | 1.53 (0.41) | 1.51 (0.40) | 1.47 (0.39) | 0.137 | 0.411 |
|  | CEMS worried subscale score | 1.55 (0.50) | 1.55 (0.46) | 1.54 (0.47) | 0.865 | 0.865 |

^1^Model 1 was adjusted for child age at follow-up only.

*Supplemental Figure 1*. Effect modifiers that showed significant differences between SQ-LNS and control groups in regions of significance analysis.


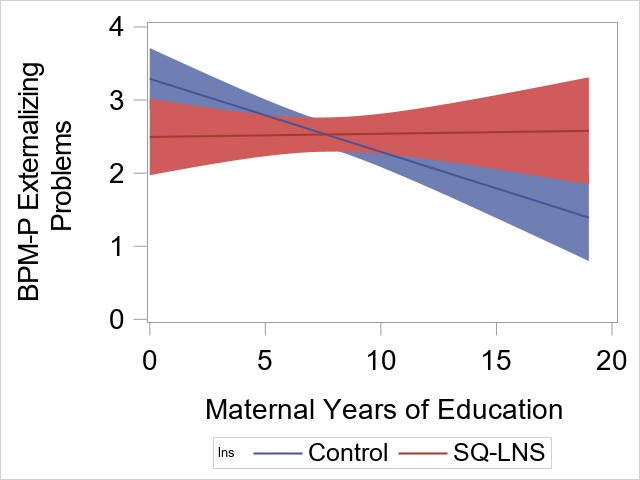

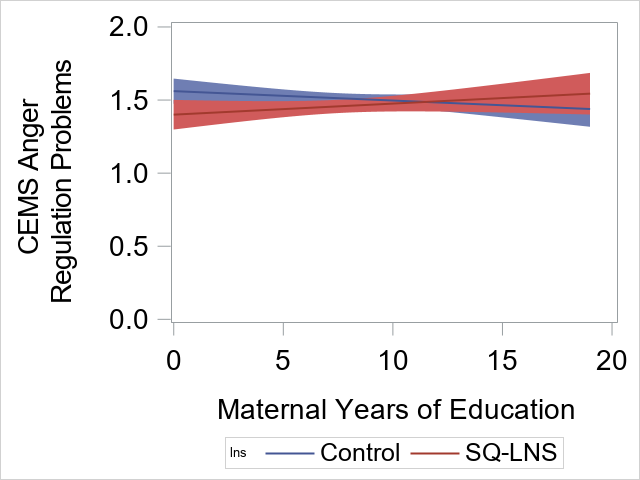


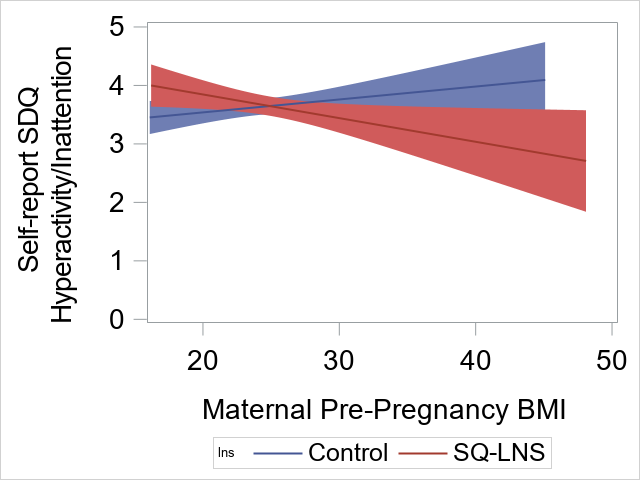

Supplement: Multimedia component1 [file mmc1.docx]
